# Frey procedure for chronic pancreatitis improves bile duct stricture

**DOI:** 10.1007/s00595-025-03148-1

**Published:** 2025-10-13

**Authors:** Hideaki Sato, Masaharu Ishida, Naohiro Hirano, Shuichiro Hayashi, Shingo Yoshimachi, Akiko Kusaka, Mitsuhiro Shimura, Shuichi Aoki, Masahiro Iseki, Daisuke Douchi, Takayuki Miura, Shimpei Maeda, Masamichi Mizuma, Kiyoshi Kume, Atsushi Masamune, Takashi Kamei, Michiaki Unno

**Affiliations:** 1 Department of Surgery, Tohoku University Graduate School of Medicine, Seiryomachi 1-1, Aobaku, Sendai 980-8574 Japan; 2 Division of Gastroenterology, Tohoku University Graduate School of Medicine, Seiryomachi 1-1, Aobaku, Sendai 980-8574 Japan

**Keywords:** Frey procedure, Chronic pancreatitis, Bile duct stricture, Biliary reconstruction, Choledochojejunostomy

## Abstract

**Purposes:**

Chronic pancreatitis (CP), a progressive inflammatory disorder characterized by pancreatic fibrosis and degeneration, often leads to common bile duct (CBD) strictures. However, the optimal management of CBD strictures remains controversial.

**Methods:**

We retrospectively analyzed 16 patients with CP and CBD strictures who underwent surgery at our institution between January 2007 and December 2024. Clinical characteristics, surgical outcomes, and impact of the Frey procedure on CBD stricture resolution were evaluated.

**Results:**

Among the 16 patients, 10 underwent biliary reconstruction and 6 underwent the Frey procedure alone. Postoperatively, CBD strictures improved in five of the six patients without biliary reconstruction (Frey alone), eliminating the need for a stent. Additionally, a long-term follow-up revealed that one patient who initially required a CBD stent experienced spontaneous improvement after the Frey procedure, suggesting its potential therapeutic effect on CBD strictures. A comparison between Frey procedure alone and biliary reconstruction cases revealed similar demographics; however, the Frey procedure alone significantly shortened operative time (*p* = 0.017).

**Conclusion:**

The Frey procedure improved CBD strictures in the majority of patients with CP by alleviating pancreatic head inflammation and fibrosis. However, biliary reconstruction may be necessary in some cases. Further studies are required to establish the optimal treatment strategies.

## Introduction

Chronic pancreatitis (CP) is a progressive inflammatory condition characterized by degeneration and fibrosis. The primary symptoms of CP are abdominal or back pain. As CP progresses, exocrine and endocrine insufficiency can lead to complications, such as malnutrition and diabetes mellitus (DM) [1]. Additionally, extensive inflammation may result in common bile duct (CBD) strictures or duodenal stenosis. CBD strictures are relatively common in CP, with an incidence ranging from 3 to 46% [2–4], although symptomatic strictures occur in approximately 10% of cases [5].

Endoscopic or surgical intervention is recommended when pain persists despite alcohol cessation, analgesics, or pancreatic enzyme therapy. Symptomatic CBD strictures and duodenal stenosis often require endoscopic or surgical management [1]. Notably, surgical intervention has been shown to be more effective than endoscopic treatment for pain control and management of CBD strictures or duodenal stenosis [6, 7].

At our institution, surgical interventions have been performed for patients with CP with intractable or recurrent pain despite medication and endoscopic treatment. The Frey procedure is described as partial coring of the head of the pancreas, but it is also a form of duodenum-preserving pancreatic head resection [8, 9]. It is a well-established surgical approach for CP, providing effective pain relief while preserving the exocrine and endocrine functions [10–12]. However, the optimal management of CBD strictures remains controversial, with treatment strategies ranging from endoscopic approaches to surgical interventions [1, 4]. In case of requiring surgical intervention, pancreaticoduodenectomy (PD) is the preferred choice if malignancy cannot be ruled out [1]. Conversely, if malignancy is not a concern, biliary reconstruction has historically been performed alongside other procedures such as the Frey procedure [13, 14].

In this study, we reviewed our surgical cases of CP with CBD strictures to evaluate the potential role of the Frey procedure in their management, comparing it to biliary reconstruction cases.

## Methods

### Ethics

The study protocol was approved by the Ethics Committee of Tohoku University Hospital (2024-1-200).

### Patients and study outline

This was a retrospective observational study. Consecutive patients with CP who underwent surgical intervention at our institution between January 2007 and December 2024 were identified using our medical information database and enrolled in the study. Clinical data, including preoperative and postoperative progress, blood test results, and imaging findings, such as computed tomography (CT), magnetic resonance cholangiopancreatography (MRCP), and endoscopic retrograde cholangiopancreatography (ERCP), were collected and analyzed. Initially, 150 patients with CP were included; however, one patient diagnosed with cancer was excluded, leaving 149 patients for the final analysis. Perioperative outcomes including patient characteristics, operative time, blood loss, postoperative hospital stay, morbidity, preoperative CBD stent placement, and the occurrence of postoperative biliary complications including cholangitis and the need for endoscopic intervention were compared between patients who underwent the Frey procedure alone and those who underwent choledochojejunostomy (CJ). Morbidity was defined as grade IIIa or higher according to the Clavien–Dindo classification [15].

### Common bile duct stricture

CBD strictures in the context of CP were comprehensively evaluated based on clinical history, liver function tests, serum CA 19-9 levels, abdominal CT scans, and cholangiography findings including MRCP and ERCP. Specifically, cases with elevated bilirubin levels, requiring endoscopic CBD drainage, or placement of a CBD stent were classified as having CBD stricture. Surgical indications for CBD strictures have been previously documented [4, 16]. Although most indications remain unchanged, recent advancements in endoscopic treatment have established endoscopic intervention as the initial approach for managing CBD strictures. Within this framework, if endoscopic intervention failed, we comprehensively evaluated the surgical indications for CP and established the following criteria: planned pancreatic surgery for pain management, symptomatic CBD strictures refractory to endoscopic treatment, history of cholangitis, and upstream dilation of the CBD. Regarding the specific indications for CBD reconstruction, following multidisciplinary conferences, biliary reconstruction has been proactively considered for patients with persistent CBD strictures lasting > 10 months, during which irreversible structural changes may have occurred, or in cases where concomitant duodenal stenosis hinders endoscopic access to the papilla.

### Operative procedures

The Frey procedure has been previously described in detail [9, 11, 12]. The Frey procedure is indicated for cases involving lesions in the pancreatic head and dilation of the main pancreatic duct. Although applicable in most CP cases, distal pancreatectomy (DP) is often combined when inflammation or calcification extends to the pancreatic tail. The combination of the Frey procedure with DP (Frey + DP) has been described in detail previously [10, 17]. Additionally, cases involving CBD strictures may require simultaneous intervention. PD is recommended in case of suspected pancreatic head cancer [1]. Biliary reconstruction is routinely performed alongside the Frey procedure in cases where pancreatic head inflammation is believed to be the primary cause of symptoms [13, 18]. However, recent evidence suggests that the Frey procedure alone can sometimes lead to symptom improvement [4, 18], prompting a shift away from routine biliary reconstruction. Consequently, our institution now follows a policy of performing the Frey procedure alone for CBD strictures due to CP, with stent removal once postoperative ERCP findings indicate an improvement in CBD strictures.

### Statistical analysis

All statistical analyses were performed with JMP Pro® (ver. 17.1.0 for Macintosh, SAS Institute Inc., Cary, NC, USA). Wilcoxon's signed-rank test was used for continuous variables, and Fisher's exact test and chi-square test were used for categorical data. Statistical significance was set at *p* < 0.05. All representative values are expressed as the median (range) unless otherwise noted.

## Results

### Characteristics of the patients

**Fig. 1:**
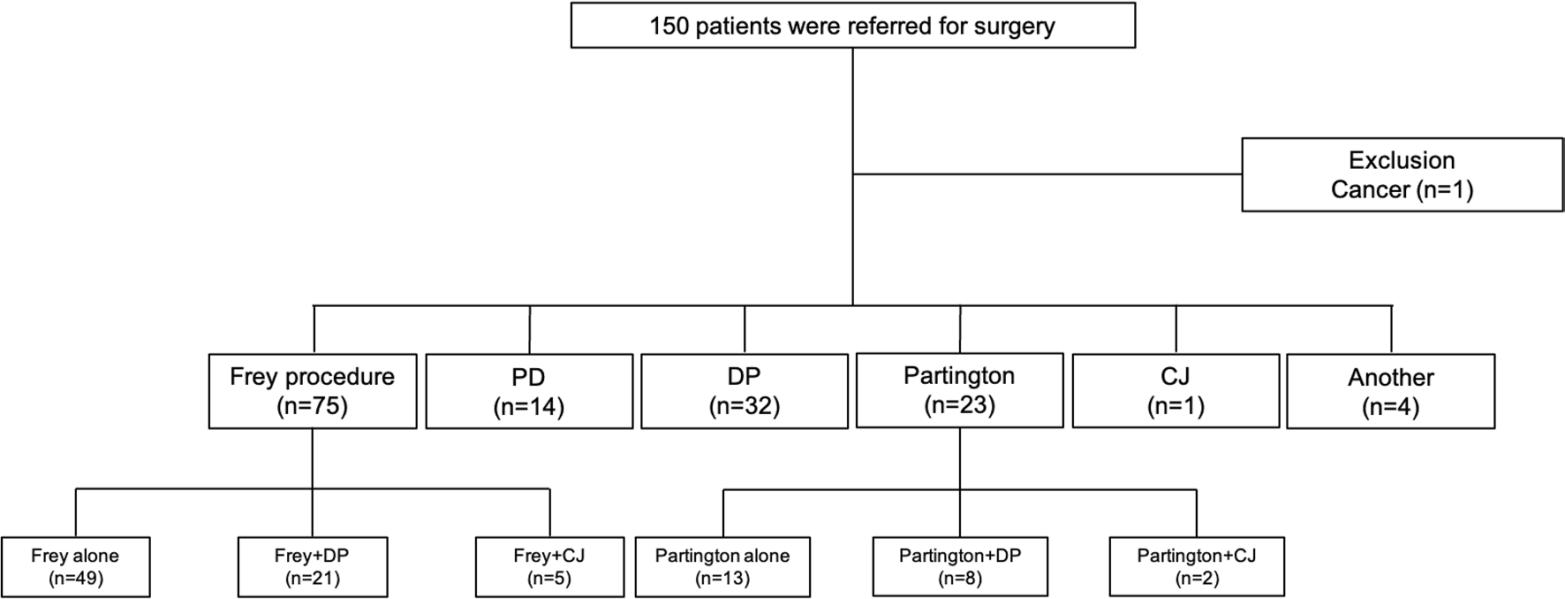
Flowchart of operative procedures for CP patients. *PD* pancreaticoduodenectomy, *DP* distal pancreatectomy, *CJ* choledochojejunostomy

The operative procedures of the 149 patients included DP (*n* = 61), PD (*n* = 14), the Frey procedure (*n* = 75), and the Partington procedure (*n* = 23), and CJ (*n* = 8). Some cases involved combined procedures, including Frey + DP (*n* = 21) and Partington + DP (*n* = 8) procedures (Fig. 1).

The characteristics of 149 patients with CP are summarized in Table 1. The age of the patients was 54 (16–81) years, with 130 males and 19 females. Preoperative DM was present in 54 patients, 121 of whom reported abdominal pain. Fifty-six patients received preoperative pancreatic enzyme replacement therapy, and the disease duration was 38.1 months. The serum albumin level, an indicator of nutritional status, was 3.7 g/dL. The operative time was 312 (111–831) min, with a blood loss of 616.5 (0–9130) mL. Morbidity was observed in 40 patients and the median postoperative hospital stay was 17 (8–306) days. CBD strictures were also observed in 16 patients. The parameters for each surgical procedure are presented separately. Some patients simultaneously underwent more than one type of surgery.

**Table 1:** Patient characteristics of all CP patients

	All cases (*n* = 149)	Frey (*n* = 75)	PD (*n* = 14)	DP (*n* = 61)	Partington (*n* = 23)
Age (years)	54 (16–81)	55 (16–77)	51 (30–76)	54 (16–80)	54 (32–81)
Sex (M:F)	130:19	64:11	13:1	52:9	20:3
Diabetes Mellitus (case)	54 (36.2%)	36 (48.0%)	2 (14.3%)	21 (34.4%)	5 (21.7%)
From onset to surgery (month)	38.1 (0.83–303)	46.4 (0.83–303)	43.1 (4.8–222)	35.0 (1.5–230)	44.0 (2.4–133)
Preoperative PERT (case)	56 (37.6%)	37 (49.3%)	4 (28.6%)	23 (37.7%)	7 (30.4%)
Endoscopic treatment (time)	1 (0–9)	2 (0–9)	0.5 (0–5)	1 (0–9)	2 (0–9)
Preoperative pain (case)	121 (81.2%)	58 (77.3%)	10 (71.4%)	50 (82.0%)	22 (95.7%)
Preoperative albumin (g/dL)	3.7 (1.4–4.7)	3.8 (1.6–4.6)	3.75 (2.7–4.5)	3.6 (1.9–4.7)	3.6 (1.9–4.7)
Operative time (min)	312 (111–831)	317 (175–527)	479.5 (334–831)	337 (170–527)	274 (170–530)
Blood loss (mL)	616.5 (0–9130)	500 (0–3460)	1415 (338–4540)	915 (24–9130)	445 (39–1915)
Post operative hospital stay (day)	17 (8–306)	16 (9–306)	23.5 (14–66)	16 (9–58)	18 (8–63)
Morbidity (case)	40 (26.8%)	21 (28.0%)	3 (21.4%)	15 (24.6%)	7 (30.4%)
CBD stricture (case)	16 (10.7%)	11 (14.7%)	2 (14.3%)	1 (1.6%)	2 (8.7%)

Morbidity was defined as grade IIIa or higher according to the Clavien-Dindo Classification. Endoscopic treatment indicated endoscopic intervention before surgery. Some cases involved combined procedures

*PD* pancreaticoduodenectomy, *DP* distal pancreatectomy, *PERT* pancreatic enzyme replacement therapy

The characteristics of the 16 patients with CBD strictures are summarized in Table 2. Of these, 13 had a CBD stent inserted preoperatively, with a stent duration of 10 months (1–24 months). Surgical interventions included PD (*n* = 2) and CJ (*n* = 8), with the following variations: Frey + CJ (*n* = 5), Partington + CJ (*n* = 2), and CJ alone (*n* = 1). PD was performed due to suspected cancer in one case, and inflammation extending to the duodenum, resulting in bleeding in the other case. The Frey procedure alone or Frey + DP was performed in six cases. Additionally, gastrojejunostomy (GJ) was performed in three cases due to duodenal stenosis. Among the eight CJ cases, one patient developed postoperative cholangitis and required hospitalization. In contrast, among the six cases in which the Frey procedure was performed alone, two required endoscopic intervention. One patient continued to require a CBD stent, while the other initially required regular CBD stent replacements but subsequently experienced resolution of the CBD stricture.

**Table 2:** Our experience with surgical treatment for CBD strictures. Characteristics of the 16 patients with CBD strictures

No	Age (year)	Sex (M:F)	Procedure	Stent (Yes/No)	Duration of stent (month)	Postoperative intervention
Case 1	52	M	Frey	Yes	3	None
Case 2	34	M	Frey + CJ	Yes	12	None
Case 3	52	M	Frey	No	None	None
Case 4	55	M	Frey + CJ	Yes	5	None
Case 5	50	F	Frey	Yes	2	None
Case 6	43	M	Frey + CJ	Yes	21	None
Case 7	42	M	Frey	Yes	12	Endoscopy and still need stent
Case 8	47	M	Frey + CJ	Yes	4	None
Case 9	58	M	Frey + DP	Yes	10	Endoscopy
Case 10	58	M	Frey + CJ + GJ	Yes	24	None
Case 11	51	M	Frey	Yes	9	None
Case 12	51	M	PD	Yes	1	None
Case 13	55	M	CJ	Yes	10	None
Case 14	48	M	PD	No	None	None
Case 15	46	M	Partington + CJ + GJ	Yes	19	None
Case 16	54	M	Partington + CJ + GJ	No	None	Cholangitis

### Case report

**Fig. 2:**
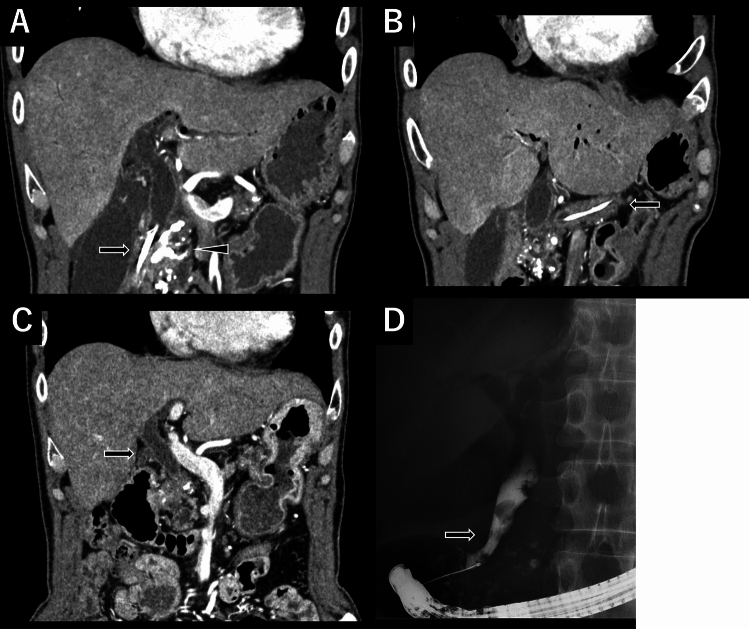
Imaging studies of a case with improvement of a CBD stricture 53 months after the Frey procedure. **A** Preoperative computed tomography (CT) revealing calcification in the pancreatic head (arrowhead) and a CBD stent in place (arrow). **B** Preoperative CT imaging showed the pancreatic duct stent in place. **C** At 53 months after surgery, the CBD stricture had resolved. **D** Although CBD dilation was still observed, the CBD stent was no longer required

We present the case of a patient with a CBD stricture that persisted for 10 months before undergoing the Frey procedure. The 58-year-old man was diagnosed with CP in 2010. Despite conservative and endoscopic treatments, pain control remained inadequate. Approximately nine years after the onset of CP, surgical intervention was performed. A CBD stricture was observed 10 months before surgery, leading to the placement of a CBD stent. Preoperative computed tomography (CT) revealed calcification in the pancreatic head, with the CBD and pancreatic duct stents in place (Fig. 2A, B). Additionally, inflammation was observed in the pancreatic tail, prompting the decision to perform Frey + DP. The operative time was 346 min, with an estimated blood loss of 365 mL. The patient experienced no major postoperative complications and was discharged on postoperative day 22. A regular follow-up with CT and a routine CBD stent exchange were conducted postoperatively. At 53 months after surgery, the CBD stricture had resolved. Although CBD dilation was still observed, a CBD stent was no longer required (Fig. 2C, D).

### Comparison between Frey procedure alone and choledochojejunostomy

The results of the comparison between the Frey procedure alone and the CJ procedure are summarized in Table 3. There were no significant differences in the clinicodemographic data between the two groups, including sex, age, CBD stent placement, and duration of CBD stent placement before surgery. Similarly, no significant differences were observed in perioperative outcomes, such as blood loss volume, postoperative morbidity, and length of hospital stay. Among the six patients who underwent the Frey procedure alone, CBD stents were placed in five, with a median stent duration of 9 (2–12) months. CBD strictures improved in five cases, eliminating the need for further stent placement; however, one patient still required a stent. In contrast, among the eight patients who underwent CJ, seven had CBD stents placed preoperatively, with a median stent duration of 12 (4–24) months. One patient developed postoperative cholangitis and required hospitalization. There were no significant differences in the duration of preoperative stent placement or frequency of postoperative intervention between the two groups. However, the operation time for the Frey procedure alone was significantly shorter (*p* = 0.017).

**Table 3:** Comparison between the Frey procedure alone and CJ

	Frey alone (6 cases)	CJ cases (8 cases)	*p* value
Age (year)	51.5 (42–58)	50.5 (34–58)	1
Sex (M:F)	5:1	8:0	0.18
Operation time (min)	297.5 (208–415)	461 (254–530)	0.017
Bleeding (mL)	370.5 (260–2005)	439 (115–1845)	1
Postoperative hospital stay (day)	22 (10–26)	14 (10–59)	0.19
Morbidity (case)	3	1	0.12
CBD stent (case)	5	7	0.83
Duration of CBD stent (month)	9 (2–12)	12 (4–24)	0.14
Postoperative intervention (case)	2	1	0.35

The characteristics of the 16 patients with CBD strictures

*CJ* choledochojejunostomy, *GJ* gastrojejunostomy

## Discussion

Surgical interventions for CP include pancreatic duct drainage procedures, such as Puestow and Partington procedures [19, 20], and resection procedures, such as PD, DP, and duodenum-preserving pancreatic head resection (Beger procedure) [21]. Pancreatic duct drainage preserves the pancreatic function, but is less effective in cases requiring control of inflammation in the pancreatic head or tail, particularly when an inflammatory mass is present [22]. Conversely, pancreatectomy effectively removes localized lesions but carries a high risk of pancreatic exocrine and endocrine insufficiency [23]. Notably, these procedures, with the exception of PD, are not indicated for CBD strictures, which may require alternative interventions. Historically, biliary reconstruction has often been performed simultaneously with other procedures such as the Frey procedure [4, 13, 14]. However, recent reports have demonstrated that CBD strictures can improve with the Frey procedure alone [4, 18]. Our findings support the potential effectiveness of this method.

CBD strictures are a well-recognized complication of CP, second only to the pseudocyst formation. They are most frequently associated with an inflammatory mass or calcification in the pancreatic head, occurring in up to 50% of patients with an inflammatory head mass [24, 25]. The reported incidence of CBD strictures in CP varies widely (2.7%–45.6%) [4], and among those requiring surgical intervention, the rate increases to 15–60% [26]. At our institution, the incidence rate was 10.7% (16 of 149 cases), potentially reflecting the increasing use of endoscopic treatment as a first-line approach.

CBD strictures in CP can result from edema, pseudocyst formation, or encasement of the CBD by fibrotic processes [4]. Although strictures due to edema or pseudocysts often resolve spontaneously, surgical intervention is traditionally recommended to prevent cholangitis and secondary biliary cirrhosis [4, 14, 18]. However, low incidence of these complications and advances in endoscopic techniques have shifted the management strategies [1, 4, 14]. Surgical options including CJ, choledochoduodenostomy, and bile duct reinsertion during the Frey procedure are still used in selected cases [14, 27]. However, these techniques carry long-term risks including cholangitis and anastomotic strictures. In our study, some patients experienced improvement in CBD strictures without requiring biliary reconstruction. This finding suggests that coring out of the pancreatic head during the Frey procedure may have a direct beneficial effect on CBD strictures. Additionally, the reduction of inflammation in the pancreatic head may resolve the associated edema and cysts, further contributing to the alleviation of CBD strictures.

A review of previous studies on the historical evolution of surgical interventions for CBD strictures in CP indicates that before the development of endoscopic intervention, surgical treatment typically involves simultaneous biliary reconstruction [6, 16]. Since 2010, four reports have documented surgical interventions for CBD strictures. Ray et al. reported surgical intervention in 41 CP cases with CBD strictures, including 25 cases treated with the Frey procedure alone and 16 cases involving biliary reconstruction including PD. Among the 25 patients treated with the Frey procedure alone, postoperative CBD strictures were observed in two cases: one was managed endoscopically, whereas the other required reoperation with CJ [4]. Rebibo et al. reported 15 cases in which the Frey procedure was performed, all of which involved simultaneous biliary reconstruction [14]. Similarly, Merdrignac et al. described a study of 29 cases treated with procedures involving biliary reconstruction [13]. In another report by Ray et al., surgical intervention was performed in 59 patients: 16 with the Frey procedure alone, 36 with the Frey procedure and simultaneous biliary reconstruction, and 7 with other forms of biliary reconstruction. Notably, CBD strictures improved in 15 of the 16 patients treated with the Frey procedure alone [18]. The data of the cases treated with the Frey procedure alone that were reported across these studies originated from a single institution [4, 18], with potential case overlap. However, when combined with cases from our institution, a total of 47 cases were identified, with postoperative CBD strictures requiring intervention in 4 cases (8.5%) and reoperation in 1 case (2.1%). While these findings suggest that CBD strictures often improve with the Frey procedure alone, no previous study has directly compared the Frey procedure alone with biliary reconstruction.

In this study, we conducted the first comparison between Frey procedure alone and biliary reconstruction, and demonstrated that the Frey procedure alone is sufficient for managing CBD strictures in CP. However, owing to small sample size, it remains unclear which patients truly do not require biliary reconstruction, making it difficult to draw definitive conclusions. Although this uncertainty may be a concern, the availability of endoscopic treatment for persistent postoperative CBD strictures helps mitigate its impact. Furthermore, as demonstrated in our study, in some cases, it was eventually possible to remove the CBD stents after surgery, even after a prolonged period, suggesting that initially forgoing biliary reconstruction may be a reasonable option. Additionally, because pancreatic surgery itself is a major surgery [28, 29], the significantly shorter operative time of the Frey procedure alone helps us to minimize invasiveness. In other words, the Frey procedure alone may be a viable surgical approach for patients with CP with CBD strictures when endoscopic intervention can be safely performed.

The present study was associated with several limitations. First, as a single-center, retrospective observational study, it is inherently subject to potential confounding factors and selection bias. Second, despite our institution being a leading center for CP treatment in Japan, the small sample size limited the generalizability of our findings. To validate these results, a prospective, multi-institutional study is warranted. Despite these limitations, this study revealed that the Frey procedure was effective in many cases of CBD strictures, supporting the findings of previous reports.

In conclusion, based on our single-center experience, while the Frey procedure was effective in resolving CBD strictures in a subset of patients with CP, the need for biliary reconstruction cannot be entirely eliminated, and further studies are warranted to identify the factors predicting successful outcomes with the Frey procedure alone. CP treatment should leverage the strengths of both endoscopic and surgical approaches, emphasizing a complementary and integrative strategy.
